# Morphological plasticity of ventral pallidal astrocytes is associated with D1 receptor-expressing terminals during heroin seeking

**DOI:** 10.1038/s41380-022-01560-4

**Published:** 2022-04-29

**Authors:** Anna Kruyer, Danielle Dixon, Ariana Angelis, Davide Amato, Peter W. Kalivas

**Affiliations:** 1 Department of Neuroscience, Medical University of South Carolina, Charleston, SC USA; 2 Department of Psychiatry and Psychotherapy, Charité - Universitätsmedizin Berlin, Berlin, Germany

(**A**) Astrocytes in the dorsolateral ventral pallidum (green) were co-labeled with terminals from nucleus accumbens D1 or D2 receptor-expressing neurons (red). Imaged astrocytes (green, **B**) were digitally rendered (**C**) and their co-registration with mCherry-labeled terminals (red) and the presynaptic marker synaptojanin 1 (SYNJ1, blue) was quantified (**D**, white; box magnified in inset). (**E**) The degree of triple co-registration in D1-Cre rats was elevated during extinction training, indicating increased astroglial proximity to D1-expressing terminals (Kruskal–Wallis = 10.38 *p* = 0.0056, **p = 0.004 Ext v. Sal using Dunn’s test). This increase was restored to control levels during cue-induced reinstatement of heroin seeking (*p* > 0.9999 Sal v. Rst using Dunn’s test). No changes were detected at synapses containing D2-neuron terminals (**D**, Kruskal–Wallis = 1.571 *p* = 0.4560). (**E**) Data shown as median, N shown in legend as cells/animals. Sal, yoked saline; Ext, extinguished; Rst, 15 min reinstated. For more information, please refer to the article by Kruyer et al. in this issue.

